# Dynamic weight bearing is an efficient and predictable method for evaluation of arthritic nociception and its pathophysiological mechanisms in mice

**DOI:** 10.1038/srep14648

**Published:** 2015-10-29

**Authors:** Andreza U. Quadros, Larissa G. Pinto, Miriam M. Fonseca, Ricardo Kusuda, Fernando Q. Cunha, Thiago M. Cunha

**Affiliations:** 1 Department of Pharmacology, Ribeirão Preto Medical School, University of São Paulo, Brazil

## Abstract

The assessment of articular nociception in experimental animals is a challenge because available methods are limited and subject to investigator influence. In an attempt to solve this problem, the purpose of this study was to establish the use of dynamic weight bearing (DWB) as a new device for evaluating joint nociception in an experimental model of antigen-induced arthritis (AIA) in mice. AIA was induced in Balb/c and C57BL/6 mice, and joint nociception was evaluated by DWB. Western Blotting and real-time PCR were used to determine protein and mRNA expression, respectively. DWB detected a dose- and time-dependent increase in joint nociception during AIA and was able to detect the dose-response effects of different classes of analgesics. Using DWB, it was possible to evaluate the participation of spinal glial cells (microglia and astrocytes) and cytokines (IL-1β and TNFα) for the genesis of joint nociception during AIA. In conclusion, the present results indicated that DWB is an effective, objective and predictable test to study both the pathophysiological mechanisms involved in arthritic nociception in mice and for evaluating novel analgesic drugs against arthritis.

Arthritis is an inflammatory articular disorder involving one or more joints and characterized by pain, swelling, joint stiffness and disability. Arthritis is a disease that predominates in the elderly, but children and young adults can also be affected. There are different forms of arthritis, such as osteoarthritis, rheumatoid arthritis, gout, septic arthritis, and others. All forms are characterized by the presence of immune cells in the joint and an increased concentration of inflammatory mediators in synovial fluid. Pain is one of the most prevalent symptoms of arthritis^1^. Pain is considered severe in 60% of patients and when combined with mechanical factors, including cartilage degradation and psychological aspects, causes moderate or severe disability in 70% of affected individuals^2^.

To better understand the pathophysiological mechanisms involved in arthritic pain, basic research studies are fundamental. However, the assessment of articular nociception in experimental animals is a challenge, especially because available methods present some limitations. For example, in almost all behavioral nociceptive tests, there is one direct investigator responsible for application of the nociceptive stimuli (mechanical, thermal) and/or visualization/quantification of the behavior/nociceptive end-point, which could be considered subjective analyses. Therefore, more objective methods with less investigator interference are required. One possible alternative to address this problem is Dynamic Weight Bearing (DWB), a device that measures the difference of weight exerted by each paw on a full-sensor floor, allowing to animals walk freely without experimenter interference. These characteristics make this method unique in evaluating the natural behavior of animals under nociceptive conditions. This was proven in models of inflammatory, neuropathic and cancer nociception in rats paws^3^, and in CFA-induced paw inflammation in mice^4^,^5^; however, no paper until now has used DWB to evaluate joint nociception.

Arthritic pain is multifactorial, complex and hard to treat. Currently, pharmacological treatment consists of non-steroidal and steroidal anti-inflammatory drugs (NSAIDs, SAIDs), selective inhibitors of cycloxigenase-2 (COX-2), disease-modifying anti-rheumatic drugs (DMARDs, e.g. methotrexate) and opioids (e.g., morphine). Immunobiological drugs, such as anti-TNF therapies, are also effective in controlling joint pain, especially in patients who are refractory to other drugs. Nevertheless, novel therapeutic options are necessary to treat non-responsive patients and avoid the side effects of other drugs, the development of which will be based on knowledge of the pathophysiological mechanisms involved in induction and maintenance of arthritic pain.

The induction and maintenance of arthritic pain might involve mechanisms occurring at different levels of the nociceptive system including peripheral (primary sensory neurons) and central sensitization (spinal and supra-spinal), neuro-immune interactions, and others^6^,^7^,^8^. Among these mechanisms, the participation of glial cells has gained importance in the last decades^9^,^10^,^11^,^12^. In fact, during pathological processes, such as peripheral inflammation or damage to sensory neurons, glial cells of the spinal cord (e.g., microglia and astrocytes) become activated and mediate the induction and maintenance of chronic inflammatory and neuropathic pain^13^,^14^,^15^,^16^. Upon activation, spinal glial cells appear to trigger the release of pro-inflammatory cytokines, including IL-1β and TNF-α, which in turn facilitate neurotransmission in spinal cord^17^,^18^,^19^,^20^. Even with this understanding, knowledge about the role of glial cells in the genesis and maintenance of arthritic pain needs to be improved to provide novel targets for controlling pain. Based on the above evidence, the purpose of the present study was to establish the use of DWB as a new method for evaluating arthritic nociception in an experimental model of antigen-induced arthritis (AIA) in mice. Furthermore, we intend to use DWB to investigate the pathophysiological mechanisms involved in the induction and maintenance of arthritic pain, focusing on the role of glial cells.

## Results

### DWB is an effective test to detect joint nociception in AIA model

**Figure 1:**
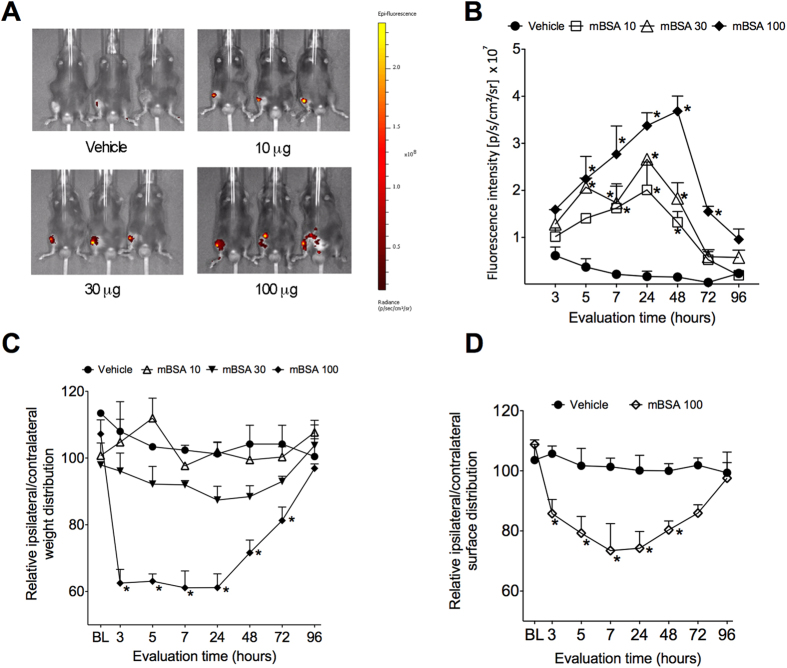
DWB was able to detect joint nociception in AIA model. Immunized Lysm-eGFP mice were challenge with i.a. with 10, 30 or 100 μg per joint of mBSA or vehicle (veh—sterile saline). (**A**) Representative fluorescence images of *in vivo* inflammation obtained in IVIS Spectrum 24 h after mBSA injection. (**B**) Quantification of mean fluorescence intensity of joints during the time course of mBSA-induced arthritis. Immunized Balb/C mice were challenge with i.a. with 10, 30 or 100 μg per joint of mBSA or vehicle (veh—sterile saline). Weight distribution (**C**) or paw surface distribution (**D**) was evaluated before and 3 up to 96 hours after mBSA injection using DWB. Data are means ± S.E.M. (n = 11). *P < 0.05 compared with vehicle group. BL (baseline).

First, joint inflammation was monitored during the course of AIA^21^,^22^ using an *in vivo* imaging system. The i.a. injection of mBSA in immunized LysM-eGFP mice produced dose- (10–100 μg/joint) and time- (1–96 h) related increases in joint fluorescence compared with the vehicle-injected group, suggesting an increase in the recruitment of leukocytes (neutrophils and macrophages) to local sites of inflammation (Fig. 1A,B).

This increase in joint inflammation was accompanied by significant changes in load distribution (Fig. 1C), as well as, in the paw surface distribution (Fig. 1D) between affected and non-affected hind limbs in a dose-dependent manner as evaluated by DWB, reflecting joint nociception. The dose of 100 μg/joint of mBSA produced the most pronounced effect, with maximal changes approximately 40% between 3 and 5 hours after challenge, and approximately 43% between 7 and 24 hours, when compared to the vehicle-injected mice (Fig. 1C). Joint nociception remained significant at 48 (33%) and 72 hours (24%) after challenge and recovered almost completely after 96 hours (9%). Additionally, AIA-induced joint nociception, evaluated by DWB, has the same profile when analyzed in C57Bl/6 mice (Supplementary Figure 1). The mBSA challenge in sham-immunized C57BL/6 mice did not produce any change in weight distribution between injected and non-injected hind limbs (Supplementary Figure 1) Finally, AIA-induced nociception was also evaluated by femur-tibial flexion reflex model using electronic von Frey (Supplementary Figure 2)^23^.

### DWB is a predictive method for analgesic drugs against arthritic pain

**Figure 2:**
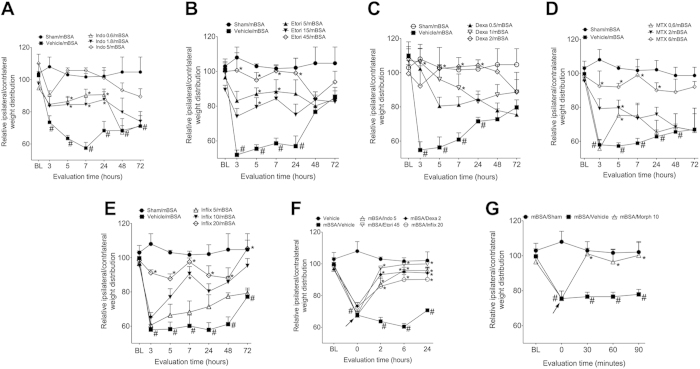
DWB presents predictivity to clinically-used analgesics in AIA model. Immunized mice were challenge i.a. with 100 μg per joint of mBSA or vehicle (veh—sterile saline). Animals were pre treated with (**A**) indomethacin (indo—0.6, 1.8 and 5 mg/kg s.c. 30 minutes before challenge), (**B**) etoricoxib (etori—5, 15 or 45 mg/kg s.c. 30 minutes before challenge), (**C**) dexamethasone (dexa—0.5, 1 or 2 mg/kg s.c. 60 minutes before challenge), (**D**) methotrexate (MTX—0.6, 2 or 6 mg/kg v.o. 5 consecutive days before challenge) or (**E**) infliximab (inflix—5, 10 or 20 mg/kg i.p. 48 hours and 60 minutes before challenge). (**F**) Animals were post treated (arrow) with indo (5 mg/kg s.c.), etori (45 mg/kg s.c.), dexa (2 mg/kg s.c.) or inflix (20 mg/kg i.p.) 6 hours after mBSA challenge and the weight distribution was evaluated 2, 6 and 24 hours after drugs administration. (**G**) Mice were post-treated (arrow) with morphine (morph—10 mg/kg s.c.) 6 hours after mBSA challenge and weight distribution was evaluated 30, 60 and 90 minutes after drug administration. Data are means ± S.E.M. (n = 5). ^#^*P* < 0.05 compared with vehicle group and *P < 0.05 compared with vehicle/mBSA group. BL (baseline).

One important characteristic of the methods used to evaluate nociception experimentally is if they are predictive for clinically-used analgesics. In this context, DWB was able to detect the recovery from changes in weight distribution caused by mBSA between affected and non-affect hind limbs when mice were pretreated with different classes of analgesic/anti-inflammatory drugs, including indomethacin (NSAID; Fig. 2A), etoricoxib (COX-2 selective inhibitor; Fig. 2B) dexamethasone (SAID, Fig. 2C), methotrexate (DMARD, Fig. 2D) and infliximab (anti-TNFα antibody; Fig. 2E). Importantly, DWB appeared to distinguish between the dose-response effects of these drugs (Fig. 2A–E). Furthermore, DWB is predictive when these drugs were administered therapeutically 6 hours after mBSA challenge (Fig. 2F). Lastly, DWB detected the opioid anti-nociceptive effect against joint nociception (Fig. 2G). It is important to mention that effective antinociceptive dose of indomethacin, etoricoxib, dexamethasone, and infliximab, but not of morphine, reduced mBSA-challenge induced an edematogenic response (Supplementary Figure 3).

### Spinal glial cells participate in joint nociception in AIA model

**Figure 3:**
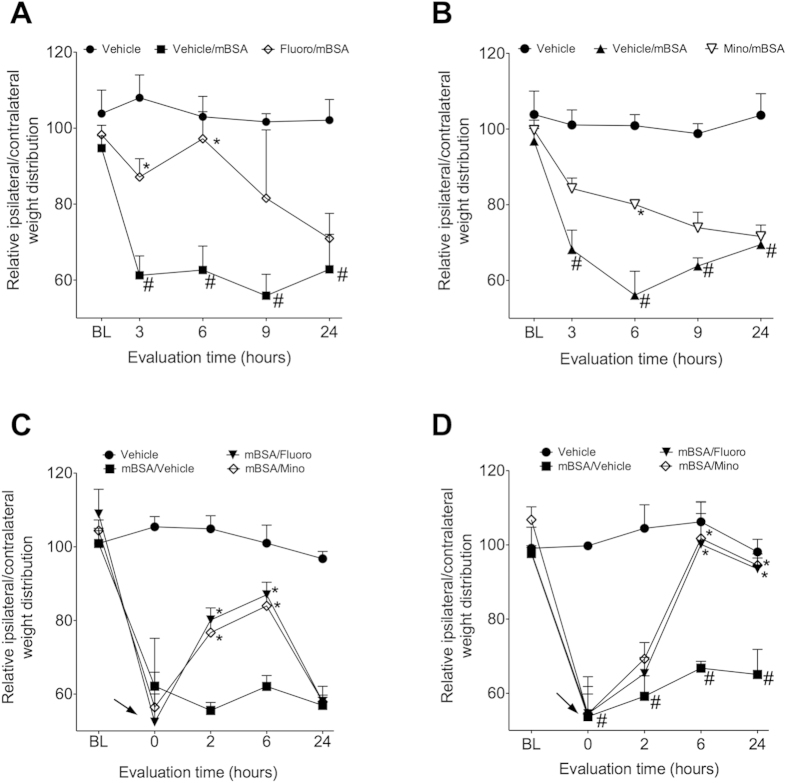
Pharmacological inhibition of spinal glial cells reduced joint nociception in AIA model. Immunized mice were challenge i.a. with 100 μg per joint of mBSA or vehicle (veh—sterile saline). Animals were pre treated with (**A**) fluorocitrate (fluoro—1 nmol/5 μL) or (**B**) minocycline (mino—1,5 μmol/5 μL) intrathecally (i.t.) 30 minutes before challenge and weight distribution was evaluated 3, 6, 9 and 24 hours after challenge. Animals were post-treated i.t. with fluoro (1 nmol) or mino (1,5 μmol) 6 (**C**) or 24 (**D**) hours after challenge. Weight distribution was evaluated 2, 6 and 24 hours after drugs administration. Data are means ± S.E.M. (n = 5). ^#^*P* < 0.05 compared with vehicle group and *P < 0.05 compared with vehicle/mBSA group. BL (baseline).

In attempt to evaluate the role of spinal microglia and astrocytes in the induction and maintenance of arthritic pain, mice were treated intrathecally with pharmacological inhibitors of astrocytes (fluorocitrate) or microglia (minocycline). First, these compounds were administered before mBSA challenge, and DWB was used to evaluate joint nociception. Pretreatment of mice with fluorocitrate or minocycline reduced changes in weight distribution caused by AIA (Fig. 3A,B). Second, fluorocitrate and minocycline reduced changes in weight distribution caused by AIA when these drugs were administered 6 (Fig. 3C) or 24 hours (Fig. 3D) after mBSA challenge.

**Figure 4:**
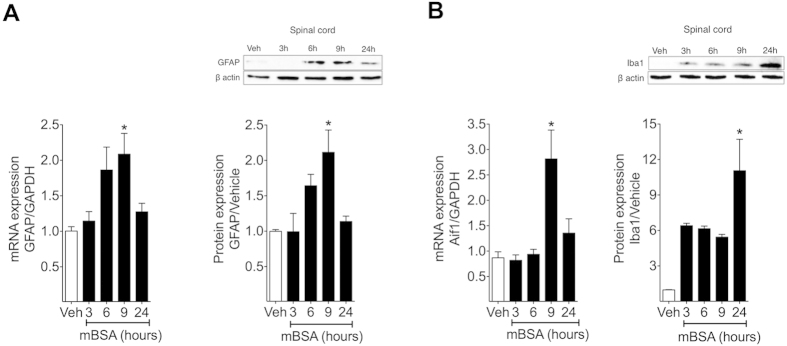
Glial cells activation markers increased in the spinal cord during AIA. Immunized mice were challenge i.a. with 100 μg per joint of mBSA or vehicle (veh-sterile saline). Spinal cord samples were collected 3, 6, 9 and 24 hours after challenge, processed and analyzed by qRT-PCR or Western Blotting (WB). (**A**) GFAP mRNA and protein expression in spinal cord were evaluated (**B**) Iba1 (Aif1 gene) mRNA and protein expression in spinal cord were evaluated. Data are means ± SEM (n = 4 to Western Blotting and 7 to PCR). *P < 0.05 compared with vehicle.

Supporting these pharmacological data, we found an increase in the mRNA and protein expression of astrocytes (GFAP, Fig. 4A) and microglia (Iba-1, Fig. 4B) activation markers in the spinal cord during AIA. GFAP protein expression was up-regulated 6 and 9 hours after mBSA challenge, whereas Iba-1 increased significantly 24h after challenge (Fig. 4A,B).

### Inhibition of spinal pro-inflammatory cytokines reverses joint nociception in an AIA model

**Figure 5:**
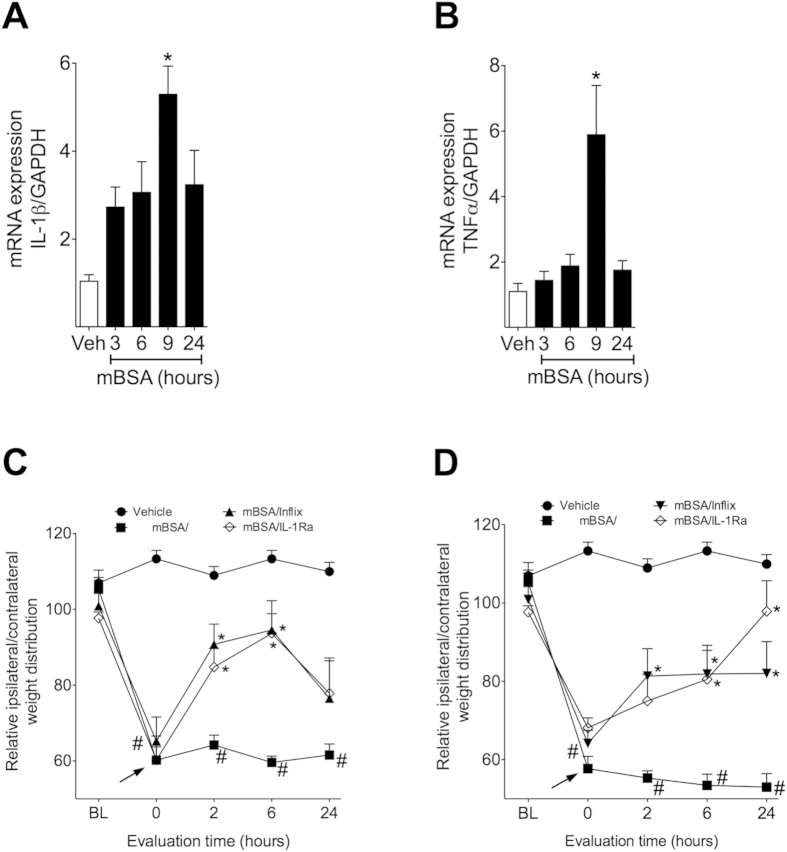
Pharmacological inhibition of cytokines reduces joint nociception in AIA model. Immunized mice were challenge i.a. with 100 μg per joint of mBSA or vehicle (veh—sterile saline). (**A**,**B**) Spinal cord samples were collected 3, 6, 9 and 24 hours after challenge, processed and analyzed by qRT-PCR. (**A**) mRNA of IL-1β and (**B**) TNF-α were normalized by GAPDH mRNA expression. Animals were post treated with IL-1Ra (300 ng/i.t.) or infliximab (100 μg/i.t.) 6 (**C**) or 24 (**D**) hours after challenge. Weight distribution was evaluated after 2, 6 and 24 hours after drugs administration. (**A**,**B**) Data are means ± SEM (n = 7) representative of two independent experiments. *P < 0.05 compared with vehicle. (**C**,**D**) Data are means ± SEM (n = 5). ^#^*P* < 0.05 compared with vehicle group and *P < 0.05 compared with vehicle/mBSA. BL (baseline).

Next, we investigated a possible mechanism by which glial cells would participate in joint nociception in an AIA model. The literature reports that in several experimental models of chronic pain, activated glia specifically release IL-1β and TNF-α at the spinal level^24^. Thus, the role of these cytokines in arthritic nociception was evaluated. The mRNA expression of these cytokines was analyzed in the spinal cord during the course of AIA. The mRNA expression of both cytokines in the spinal cord peaked at 9 hours after i.a. injection of mBSA (Fig. 5A,B). To verify the functionality of IL-1β and TNF-α in the maintenance of joint nociception, mice were treated intrathecally with IL-1 receptor antagonist (IL-1ra) or infliximab 6 or 24 hours after mBSA challenge. Both treatments reduced joint nociception (Fig. 5C,D). The intrathecal administration of IL-1ra in a dose (300 ng/5 μL i.t.) and time that inhibits AIA-induced nociception did not change AIA-induced edematogenic response (Supplementary Figure 4). Taken together, these data showed that spinal glial cells are important for the genesis and maintenance of articular pain in an AIA model, most likely by releasing IL-1β and TNF-α at the spinal level.

## Discussion

An experimental model of pathological pain relies on effective methods to evaluate its parameters. It is a challenge to assess joint function when the responses exhibited by the animal are difficult to interpret and require extensive training for the experimenter. Furthermore, the correlation between behavioral responses observed in pre-clinical trials and clinical responses is unsatisfactory. Proof of this is that approximately 80% of prototype analgesic drugs tested in humans failed in phase III^25^. Thus, establishing new criteria for animal tests is essential for the development of a novel pharmacological therapy to control pain. In this context, this work provides the standardization of DWB for evaluation of articular nociception in an experimental model of arthritis.

DWB has shown to be a sensitive and predictive tool for the assessment of joint nociception in an experimental model of arthritis. DWB discriminates between different doses of antigen-induced joint nociception and shows able to detect the dose-response effects of different classes of clinically anti-inflammatory and analgesic drugs used for arthritic pain. Compared to other methods, DWB offers a major advantage by eliminating interference from the experimenter, making it a unique, objective and differential test in the study of articular nociception. Moreover, the longer observation time of animals in DWB (5 minutes) compared to static weight bearing (5 to 10 seconds)^26^ is important for improved and more reliable evaluation. Moreover, the freedom of movement by the animal reduces the possibility of stress-induced analgesia^27^. Furthermore, the possibility of mice to execute the exploratory behavior, specifically rearing behavior, improves the evaluation of joint nociception by requiring articular force, which is reduced during hyperalgesia. In addition to arthritic pain, DWB has already been shown to be effective in detecting paw nociception in models of inflammatory, neuropathic and cancer pain in rats and CFA-induced paw inflammation in mice^3^,^4^,^5^.

Although the evidence described above indicate that DWB would be a more suitable method for joint pain evaluation, some points should be considered when choosing DWB. As the method evaluates the difference between the weights exerted by each limb of the animal, it is crucial that the experimental model of arthritis be monoarticular. Thus, DWB would not be an option to evaluate joint pain in experimental models such as collagen-induced arthritis (CIA) and collagen antibody-induced arthritis (CAIA), which are the well-accepted models of rheumatoid arthritis^28^. Another restriction of DWB is that the apparatus only allows the acquisition of data from one mouse per period and analyzing acquired data is time-consuming.

Another important question arising from our work is the following: what is the clinical symptom evaluated by DWB, hyperalgesia or spontaneous nociception? Important information that can be extracted from DWB analyses is the surface of the paw that touches the floor of apparatus (in mm^2^) when the animal walks. Interestingly, we found that this parameter is reduced in the affected hind limb in AIA mice (Fig. 1D), suggesting that mice under AIA might be guarding their paws. Importantly, when compared with femur-tibial flexion reflex model^23^, which is believed to measure joint hyperalgesia, DWB seems to be less sensitive. Thus, while DWB might detect joint hyperalgesia triggered by mice body weight, it is plausible that DWB is might detecting spontaneous nociception, which is probably caused by a higher inflammatory stimulus.

Since 1991, when Garrison proposed the involvement of glial cells in the genesis of pathological pain, several studies have emerged showing their role in several models of inflammatory, post-surgical and, especially, neuropathic pain^7^,^8^,^9^. However, there are few studies that investigate the participation of spinal glial cells in the genesis of arthritic pain, especially in AIA. We sought to investigate the involvement of spinal glial cells in joint nociception during AIA, using DWB. It was also important to evaluate whether DWB might be a useful tool in the investigation of pathophysiological mechanisms involved in arthritic pain. In this context, pharmacological inhibition of astrocytes and microglia with fluorocitrate and minocycline, respectively, were shown to be effective in reducing AIA-induced joint nociception. Whereas fluorocitrate in the tested doses is an inhibitor of the aconitase enzyme, which plays a fundamental role in the metabolism of astrocytes^25^, minocycline is a second-generation tetracycline, which has been shown to inhibit microglial cells activation^29^. Interestingly, fluorocitrate and minocycline were effective in reducing joint nociception when they were injected before and even 24 hours after AIA induction. These results might indicate that spinal glial cells are involved in the induction and maintenance phases of joint pain.

The involvement of glial cells in joint nociception implies they are activated and release proinflammatory products. Glial activation can be evaluated by analyzing the expression of some markers, including GFAP in astrocytes and Iba1 in microglial cells^30^. Both qRT-PCR and Western blotting confirmed that glial cells of the spinal cord are activated during our AIA model. Interestingly, our results showed that GFAP mRNA and protein expression precedes Iba1 expression, suggesting a previous astrocytic activation regarding microglia. This profile differs from those obtained in models of neuropathic pain, in which microglial activation appears to precede astrocytic activity^30^,^31^,^32^. Thus, the profile of glial cell activation may be dependent on the triggering stimulus and pathology. Although molecular data have revealed this glial cells activation profile, the participation of glial cells in arthritic nociception over the time course evaluated by pharmacological treatments appears to differ. In fact, minocycline and fluorocitrate were effective in reducing joint nociception even when microglia and astrocytes activation markers are not up-regulated. In fact, the lack of expression of glial activation markers did not indicate that glial cells were not active and releasing pro-inflammatory products. Another important point to consider in this discussion is that the intrathecal route of administration allows distribution of the drug in both the spinal cord as well as in the DRG^33^. Thus, we could not exclude the possibility that fluorocitrate could be inhibiting the activity of satellite glial cells in the DRGs, which are described as important for the maintenance of inflammatory and neuropathic pain^10^,^34^.

Once spinal glial cells are activated during the AIA model, their participation in the induction and maintenance phases of joint nociception might be through the production of pro-inflammatory cytokines such as IL-1β and TNFα^35^,^36^. In this context, the role of the most important cytokines produced by spinal glial cells in AIA-induced joint nociception was evaluated using DWB. Corroborating this hypothesis, there was an up-regulation in the expression of IL-1β and TNFα mRNA in the spinal cord of mice subjected to AIA, a result that is temporally compatible with glial cell activation. Interestingly, the reduction in joint nociception when IL-1Ra and infliximab were administered after mBSA challenge was relatively similar to that obtained when the glial cells were inhibited, reinforcing the idea that glial cells participate in joint nociception through the release of IL-1β and TNFα. In agreement, spinal glial cells are the main source of IL-1β and TNFα in other models of chronic pain^37^,^38^,^39^,^40^ and inhibition of glial cells activation reduced the production of IL-1β and TNFα after peripheral inflammation or nerve injury^41^,^42^.

It is noteworthy that the inhibition of glial cells, IL-1β, and TNFα at the induction (6 h after mBSA challenge) and maintenance phases (24 h after mBSA) were shown to be effective in reducing joint nociception. Nevertheless, the effect of glial cells, IL-1β and TNFα blockage at 6 h after mBSA administration is not maintained after 24 h. This result is most likely explained by the pharmacokinetic profile of the inhibitors given that when they were administered 24 h after challenge, the effect was restored. Furthermore, these results also suggest that spinal activation of glial cells and the release of IL-1β and TNFα, at later time points, maintains joint nociception. These events could be driven by peripheral inflammation in a mechanism dependent on the enhancement of peripheral neuronal activity^43^. Remarkably, when cytokine inhibitors were administered 24 h after AIA induction, the effect was still present at 24 h after drug administration. Although the explanation is not totally clear, it plausible that at this time point (48 h after mBSA challenge), there is no remaining peripheral input into the spinal cord and joint nociception could not be reestablished.

In summary, the present results indicate that DWB is an objective and sensitive method for assessing nociception in experimental models of arthritis. Furthermore, our results indicate that DWB can be used to assess the efficacy of clinically used analgesic drugs for joint pain and may be of essential utility in the preclinical development of novel analgesic drugs. Lastly, it is plausible to suggest that DWB is a useful tool for investigating the pathophysiological mechanisms involved in arthritic pain, including those occurring at the spinal cord level.

## Material and Methods

### Animals

The experiments were performed using male Balb/C and C57BlL6 mice or LysM-eGFP C57BL/6 mice (20–25 g), which carrying a knock-in mutation for enhanced green fluorescent protein (eGFP) in the lysozyme M-locus^44^. These transgenic mice are used to monitor the traffic of macrophage and neutrophils to inflammatory sites^45^,^46^. The animals were maintained in temperature-controlled rooms (22–25 °C) and given water and food *ad libitum*. Animal care and handling procedures were in accordance with the guidelines of the International Association for Study of Pain (IASP) and with the approval of the Animal Ethics Committee of the Ribeirao Preto Medical School, University of Sao Paulo (115/2011).

### Drugs

The following materials were obtained from the sources indicated: methylated albumin from bovine serum (mBSA), complete Freund’s adjuvant (CFA), indomethacin, dexamethasone, morphine, fluorocitric acid/flurocitrate and minocycline were obtained from Sigma–Aldrich (St. Louis, Missouri, USA). Methotrexate was prepare from pill milling of commercial med METREXATO® 2,5 mg (Blausiegel, lot 1004051). Etoricoxib was prepared from ARCOXIA® 120 mg, coated pills (Merck Sharp & Done, lot JA021) and infliximab from REMICADE® 100 mg, lyophilized powder for intravenous injection, (Schering Plough, lot L0RMKA85301). IL-1 receptor antagonist (IL-1ra) was gentle provided by Dr. Stephen Poole, from the National Institute for Biological Standards and Control (South Mimms, Hertfordshire, UK). The drugs were diluted in sterile saline, except indomethacin that was diluted in Tris buffer 1.5 M pH 8.8 and fluorocitrate was dissolved initially in 2.0 M HCl and then diluted in sterile, endotoxin-free 10 mM PBS, pH 7.4^47^.

### Induction of Antigen-Induced Arthritis (AIA)

Mice were immunized as described previously^20^,^21^. Briefly, the mice were sensitized with 500 μg of methylated bovine serum albumin (mBSA) in 0.2 mL of an emulsion containing 0.1 mL saline and 0.1 mL CFA (1 mg/mL of inactive *Mycobacterium tuberculosis*) and given by subcutaneous (s.c.) injection on day 0. The mice were boosted with the same preparation on day 7. Sham-immunized mice were given similar injections but without the antigen (mBSA—data no show). Twenty-one days after the initial injection, arthritis was induced in the immunized animals by intraarticular (i.a.) injection of mBSA (10, 30, 100 μg) dissolved in 10 μL of saline into the right femur–tibial joint.

### Dynamic Weight Bearing (DWB)

Articular nociception was evaluated using a DWB apparatus (Bioseb, France). Briefly, the device consists of a small Plexiglas chamber (11.0 × 19.7 × 11.0 cm) with floor sensors containing pressure transducers. The system uses software that records, in grams, the average weight that each limb exerts on the floor, without any interference of analyzer. For testing, the mouse was placed in the chamber and allowed to move freely within the apparatus for period of 5 minutes. A camera was pointed at the side of the enclosure to assist with data analysis. All movements were filmed and validated by the experimenter in accordance with the position of the mouse on the device, indicating which paw corresponded to the set of pixels recognized by the sensors: right or left paw. The DWB software gives data about weight (in grams) or area (in mm^2^) of paw, which touch the floor. Testicles and tail were excluded from the analysis. The animals were subjected to the test without previously adaptation, since exploratory movements improve data capture. The results were expressed as percentage weight or area of the ipsilateral hind paw (right) and contralateral paw (left).

### Dorsal flexion of the femur-tibial joint: Assessment by a modified electronic pressure-meter test for mice

The articular nociception of the femur-tibial joint was also evaluated using a previous method with modification^22^,^23^. In a quiet room, mice were placed in acrylic cages (12 × 10 × 17 cm high) with a wire grid floor 15–30 min before testing for environmental adaptation. Stimulations were performed only when animals were quiet, did not display exploratory movements or defecation, and were not resting on their paws. In these experiments, an electronic pressure-meter was used. It consists of a hand-held force transducer fitted with a polypropylene tip (IITC Inc., Life Science Instruments, Woodland Hills, CA, USA). For this model, a large tip (4.15 mm^2^) was adapted to the probe. An increasing perpendicular force was applied to the central area of the plantar surface of the hind paw to induce flexion of the femur-tibial joint followed by paw withdrawal. A tilted mirror below the grid provided a clear view of the hind paw. The electronic pressure-meter apparatus automatically recorded the intensity of the force applied when the paw was withdrawn. The test was repeated until three subsequently consistent measurements (i.e. the variation among these measurements was less than 1 g) were obtained. The flexion-elicited mechanical threshold was expressed in grams (g).

### Fluorescence imaging system

Evaluation of inflammation provoked by i.a. injection of mBSA in immunized mice also was analyzed by *in vivo* real time fluorescent system IVIS Spectrum (Caliper Life Science, Hopkinton, Massachusetts, USA). Immunized LysM-eGFP mice received 10, 30 or 100 μg/joint of mBSA and were submitted to IVIS 3, 6, 9, 24, 48, 72 and 96 hours after challenge. The ROI was measured under the assistance of Living Image software, and the data were represented as average of intensity of fluorescence.

### Intrathecal injection

Intrathecal injections (5 μL) were made under anesthesia with 2% isoflurane, with a 30G needle, in a shaved area correspondent to L5/L6. The local was considered correct when, after insertion of needle, mice presented a tail reflex^48^.

### Pharmacological treatments protocols

In order to establish pharmacological predictivity of DWB in experimental arthritis, mice were treated with four different classes of clinically-used analgesic drugs: a non-steroidal anti-inflammatory drugs (NSAIDs; non-selective cyclooxygenase inhibitor; COX)—indomethacin (0.6, 1.8 or 5 mg/kg, s.c. 30 minutes before or 5 mg/kg, s.c. 6 h after the i.a. challenge with mBSA); a selective inhibitor of COX-2—etoricoxib (5, 15 or 45 mg/kg, s.c. 30 minutes before or 45 mg/kg, s.c. 6 h after of i.a. challenge with mBSA); a steroidal anti-inflammatory drug (SAID), dexamethasone (0.5, 1 or 2 mg/kg, s.c. 60 minutes before or 2 mg/kg, s.c. 6 h after the i.a. challenge with mBSA), a disease-modifying anti-rheumatic drug (DMARD), methotrexate (0.6, 2 and 6 mg/kg, for 5 consecutive days before i.a. challenge with mBSA, v.o.) and an immunobiological drug, anti-TNFα antibody—infliximab (5, 10 or 20 mg/kg, i.p. 48 hours and 60 minutes before or 20 mg/kg, i.p. 6 h after the i.a. challenge with mBSA). Arthritic mice were also post-treated with morphine 10 mg/kg s.c. 6 hour hours after i.a. challenge.

The joint nociception was evaluated using the DWB in the animals as follows: a) pre-treatment protocols: at 3, 5, 7, 24, 48 and 72 hours after i.a. mBSA challenge; and b) post-treatment protocols: at 6 hours after the i.a. challenge with mBSA and 2, 4 and 6 hours after drug treatment; c) morphine post-treatment: at 6 hours after the i.a. challenge with mBSA and 30, 60 and 90 minutes after morphine treatment.

### Pharmacological inhibition of glial cells

To investigate the role of spinal glial cells in arthritic pain, mice received pre- or post-treatments with fluorocitrate (1 nmol/5 μL, intrathecal—i.t.) and minocycline (1.5 μmol/5 μL, i.t.), two inhibitors of astrocytes and microglia, respectively. Both drugs were administered 30 minutes before i.a. challenge with mBSA or post-treated 6 or 24 hours after the i.a. injection with mBSA. The joint nociception in pre-treated groups was evaluated 3, 6, 9 or 24 hours after challenge. In the post-treated groups, joint nociception was determined 6 or 24 hours after mBSA challenge and 2, 6 and 24 hours after drugs treatments.

### Pharmacological inhibition of spinal pro-inflammatory cytokines

In order to investigate the participation of spinal IL-1β and TNF-α in joint nociception during AIA, mice were post-treated intrathecally with drugs that prevent the activity of these cytokines: IL-1ra (300 ng/5 μL i.t.) or infliximab (anti-TNF antibody; 100 μg/5 μL i.t.) 6 or 24 hours after the i.a. challenge. The joint nociception was determined 6 or 24 hours after mBSA challenge and 2, 6 and 24 hours after drugs treatments.

### Joint swelling (oedema)

Immunized Balb/C mice received the same pharmacological treatments with anti-inflammatory drugs or IL-1ra, as described previously. Before the i.a. challenge with 100 μg of mBSA, thickness of the femur-tibial joint was measured with a digital caliper, The same procedure was repeated 3, 6, 9, 24, 48 and 72 hours after challenge. Results are expressed as the difference between final and initial value (delta), in mm.

### Gene expression by qRT-PCR

The mRNA expression of GFAP (astrocytes activation marker), Aif1 (microglia activation marker), IL-1β and TNF-α were evaluated by real time PCR in spinal cord samples. Briefly, mice previously immunized were anesthetized with isoflurane 2% and sacrificed by decapitation 3, 6, 9 or 24 hours after i.a. challenged with mBSA (100 μg/ i.a.) challenge. After laminectomy, the ipsilateral spinal cord corresponding to L3, L4, L5 and L6 segment were collected. Total RNA from spinal cord was extracted using the Trizol® reagent (Invitrogen Life Technologies Corporation, Carlsbad, California, USA) according to the directions supplied by the manufacturer. The purity of total RNA was measured with a GeneQuant spectrophotometer (Amersham Biosciences Corporation, Piscataway, New Jersey, USA). The wavelength absorption ratio (260/280 nm) was between 1.8 and 2.0 for all preparations. Reverse transcription of total RNA to cDNA was carried out by reverse transcriptase Pre-Improm II (Promega, Madison, Wisconsin, USA). Real-time quantitative PCR analysis was performed in Viia7 Sequence Detection System using the SYBR-green fluorescence system (Applied Biosystems, Warrington, UK). RT-PCR was performed in 7 μL reaction volume and carried out with the following cycling parameters: Initial heating at 95 °C (10 min) followed by 40 cycles of 94 °C (1 min), 56 °C (1 min) and 72 °C (2 min). Melting curve analysis was performed (65–95 °C) to verify the amplification of a single product. Samples with more than one peak were excluded. The data were analyzed according to the comparative cycle threshold (CT) method. Primers pairs for mouse GFAP, Aif1, IL-1β, TNF-α and GAPDH were as follows:

GFAP forward: 5′—TCTAAGGGAGAGCTGGCAGGGCT—3′

GFAP reverse: 5′—AGGGCGAAGAAAACCGCATCACC—3′

Aif1 forward: 5′—TGAGGAGCCATGAGCCAAAG—3′

Aif1 reverse: 5′—GCTTCAAGTTTGGACGCCAG—3′

IL-1β forward: 5′—TGACAGTGATGAGAATGACCTGTTC—3′

IL-1β reverse: 5′—TTGGAAGCAGCCCTTCATCT—3′

TNF-α forward: 5′—AGGGATGAGAAGTTCCCAAATG—3′

TNF-α reverse: 5′—GGCTTGTCACTCGAATTTTGAGA—3′

GAPDH forward: 5′—CATCTTCTTGTGCAGTGCCA—3′

GAPDH reverse: 5′—CGGCCAAATCCGTTCAC—3′

### Western Blotting

At indicated times after i.a. injection of stimuli (mBSA 100 μg), mice were terminally anesthetized and the ipsilateral spinal cord was collected and homogenized in Rippa® buffer (Sigma–Aldrich, St. Louis, Missouri, USA) containing proteases inhibitors (Complete®- Roche Diagnostics, Indianapolis, USA). The protein concentration of the lysate was determined using Bradford colorimetric assay. The protein samples were separated on SDS/PAGE gels (12% for GFAP and 15% for Iba-1), and transferred to nitrocellulose membranes (Amersham Pharmacia Biotech, Little Chalfont, UK). The membranes were incubated with primary antibodies against GFAP (1:500; Millipore, Billerica, MA, EUA), Iba-1 (1:200; Wako Chemicals, Osaka, Japan) and β-actin (1:1,000 Millipore, Billerica, Massachusets, EUA) overnight at 4 °C, and then incubated for 3 hours at room temperature with an HRP-conjugated secondary antibodies, anti-mouse (1:3,000) for GFAP, anti-rabbit 1:10,000 for Iba-1 and anti-mouse 1:3,000 for β-actin. The blots were visualized in an ECL solution (Amersham Pharmacia Biotech, Little Chalfont, UK) and exposed in a ChemiDoc MP Imaging System (Bio-Rad Laboratories, Hercules, California, USA).

### Statistical analysis

Data are reported as the means ± SEM and are representative of two separate experiments. Two-way ANOVA was used to compare the groups and doses at the different times (curves) when the responses (nociception or fluorescence) were measured after the stimulus injection. The analyzed factors were the treatments, the time, and the time versus treatment interaction. The normality of data was analyzed by D’Agostino and Pearson test, which confirm the parametric distribution of data. If there was a significant time versus treatment interaction, one-way ANOVA followed by Bonferroni’s t-test was performed for each time. Alternatively, if the responses (nociception, protein expression, mRNA expression) were measured only once after the stimulus injection, the differences between responses were evaluated by one-way ANOVA followed by Bonferroni’s t-test (for three or more groups), comparing all pairs of columns. P values less than 0.05 were considered significant. Statistical analysis was performed with GraphPad Prism (GraphPad Software, San Diego, CA, USA).

## Additional Information

**How to cite this article**: Quadros, A. U. *et al.* Dynamic weight bearing is an efficient and predictable method for evaluation of arthritic nociception and its pathophysiological mechanisms in mice. *Sci. Rep.* **5**, 14648; doi: 10.1038/srep14648 (2015).

## Supplementary Material

Supplementary Information
