# Supplementary material for: Dynamic weight bearing is an efficient and predictable method for evaluation of arthritic nociception and its pathophysiological mechanisms in mice
Source: Sci Rep. 2015 Oct 29;5:14648. doi: 10.1038/srep14648 (PMC4625149; doi:10.1038/srep14648)
Supplement: Supplementary Information [file srep14648-s1.pdf]

Supplementary information

Dynamic weight bearing is an efficient and predictable method for evaluation of arthritic nociception and its pathophysiological mechanisms in mice

Andreza U. Quadros[1], Larissa G. Pinto[1], Miriam D. Fonseca[1], Ricardo Kusuda[1], Fernando Q. Cunha[1], Thiago M. Cunha[1*].

[1] Department of Pharmacology, Ribeirão Preto Medical School, University of São Paulo, Brazil

Corresponding author: Thiago Mattar Cunha, PhD, Ribeirao Preto Medical School, University of Sao Paulo, Bandeirantes Avenue, 3900 –14049-900, Ribeirão Preto, São Paulo, Brazil. Phone +55 16 3602-0199. E-mail: thicunha@fmrp.usp.br

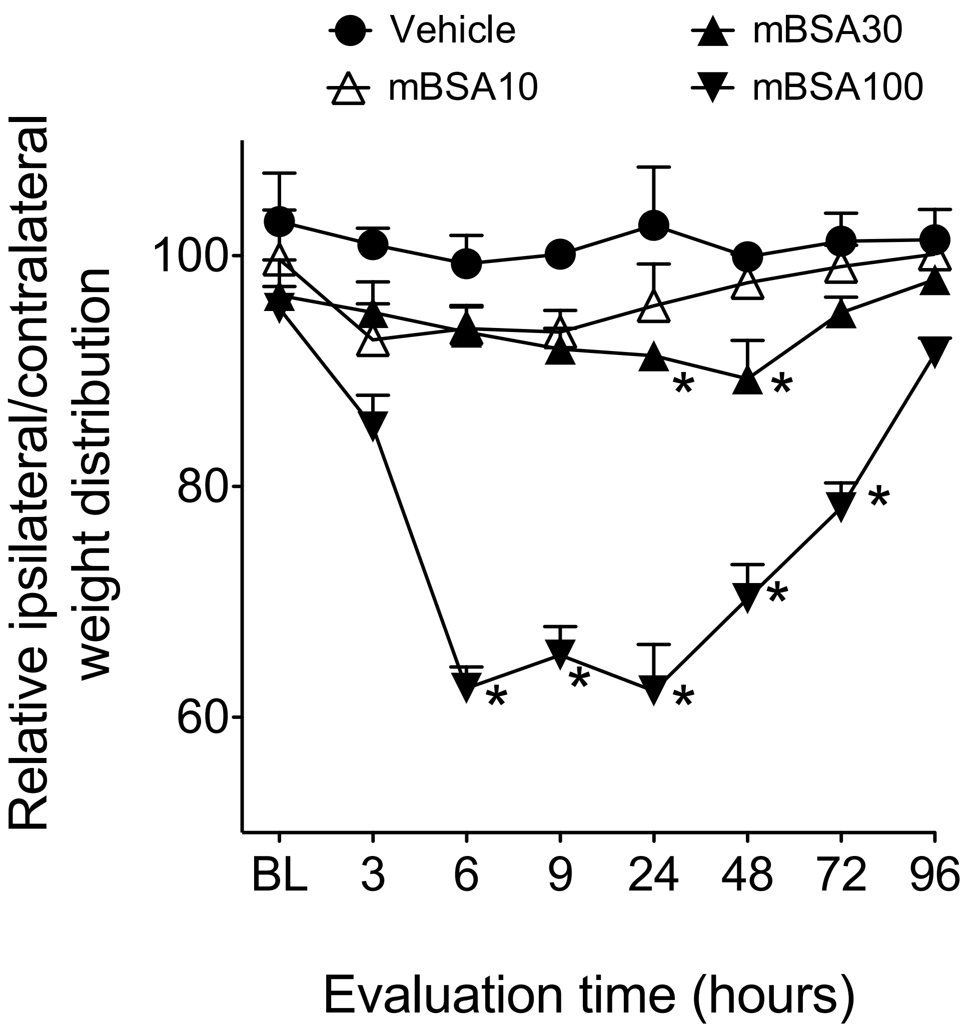

Supplementary Figure 1- DWB was able to detect joint nociception in C57BL/6 mice during AIA model. Immunized C57BL/6 male mice were challenge i.a. with 10, 30 or 100 μg per joint of mBSA or vehicle (veh - sterile saline). Weight distribution was evaluated before and 3 up to 96 hours after mBSA injection using DWB. Data are the means ± S.E.M (n=5). **P* < 0.05 compared with vehicle group. BL (baseline).

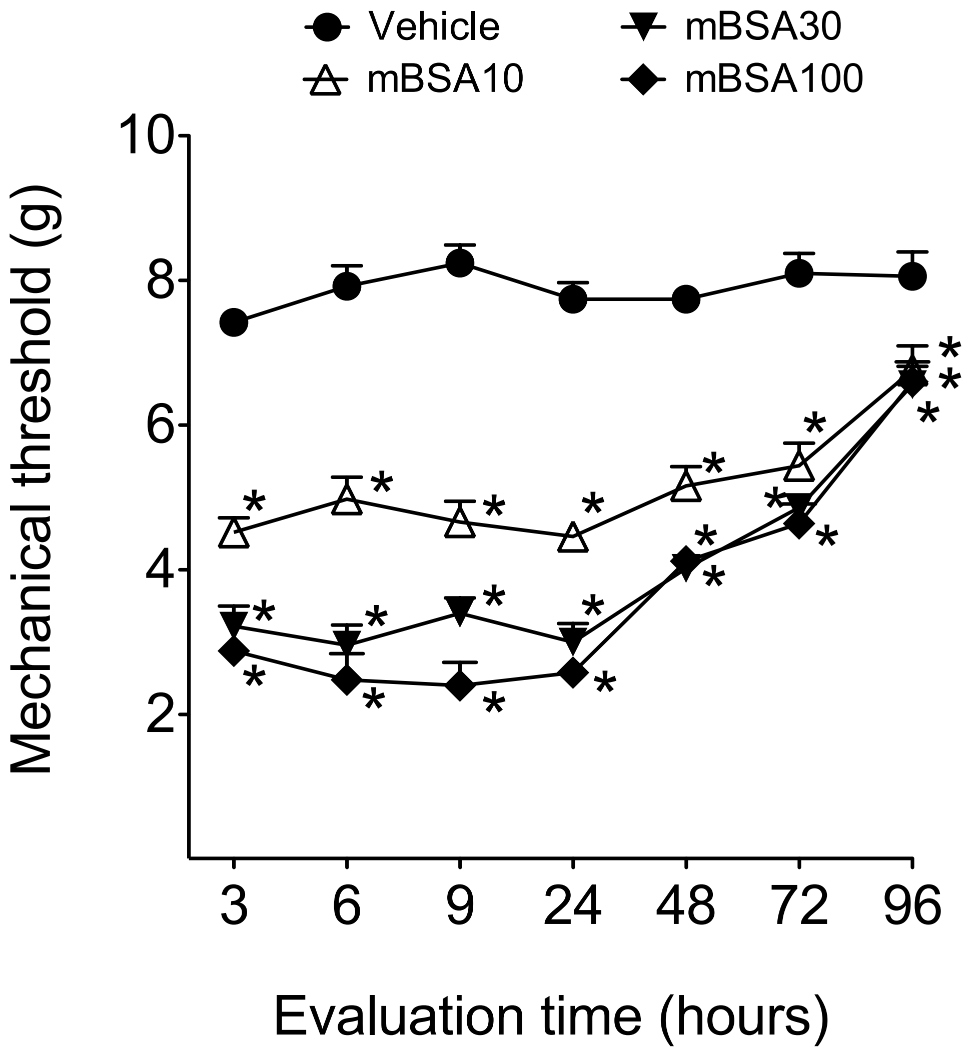

Supplementary Figure 2- Mechanical nociceptive threshold in Balb/C mice during AIA. Immunized Balb/C male mice were challenged with i.a. with 10, 30 or 100 µg per joint of mBSA or vehicle (veh - sterile saline). Mechanical threshold was evaluated before and 3 up to 96 hours after mBSA injection using electronic von Frey. Data are the means ± SEM (n=5). *$P < 0.05$ compared with vehicle group.

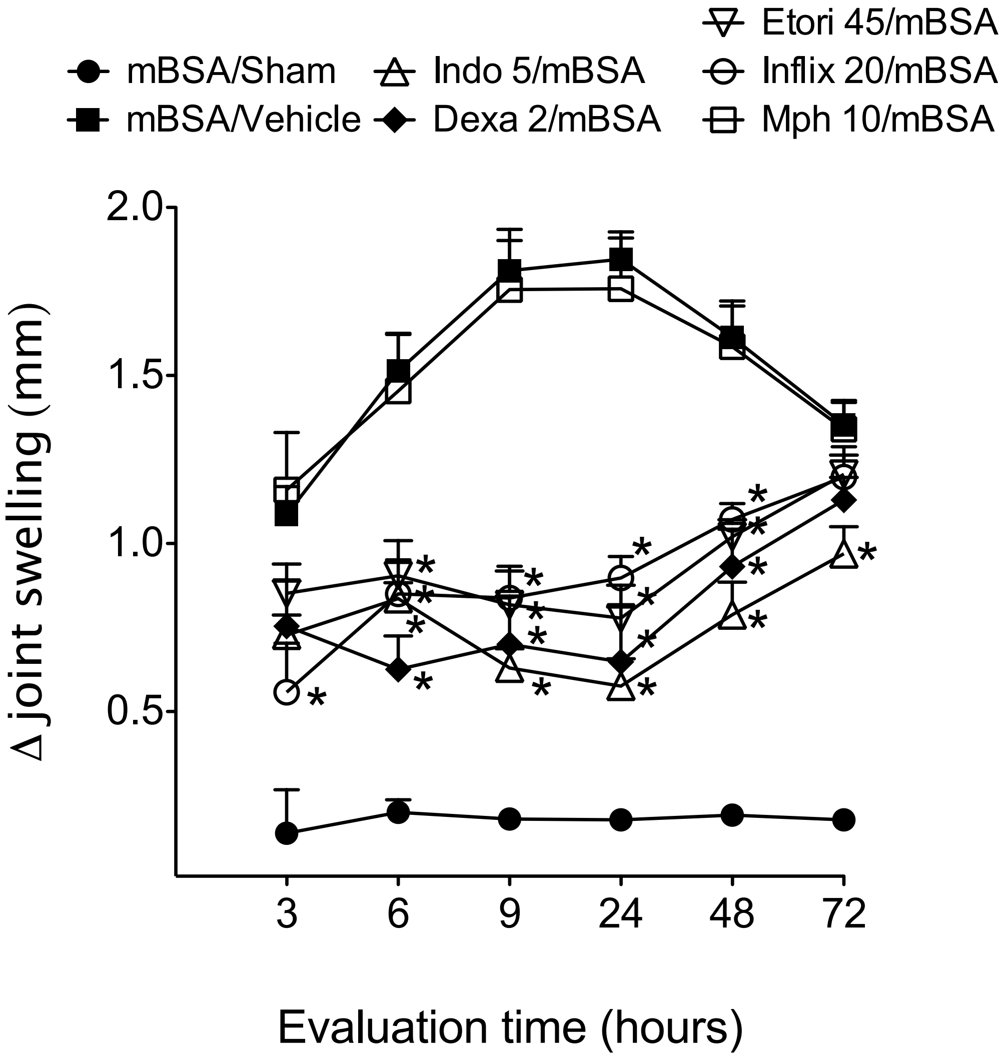

Supplementary Figure 3- Effect of classical anti inflammatory drugs and morphine on joint swelling (oedema) in Balb/C mice during AIA model. Immunized mice were challenge i.a. with 100 µg per joint of mBSA or vehicle (veh - sterile saline). Animals were pre treated with indomethacin (indo – 5 mg/kg s.c. 30 minutes before challenge), etoricoxib (etori – 45 mg/kg s.c. 30 minutes before challenge), dexamethasone (dexa – 2 mg/kg s.c. 60 minutes before challenge), infliximab (inflix –20 mg/kg i.p. 48 hours and 60 minutes before challenge) or morphine (mph - 10 mg/kg s.c. 3 h after challenge). Joint swelling was measure with a digital caliper in animals under anesthesia (isoflurane 2%), before and 3 up to 72 hours after challenge. Data are means of delta (final measure subtracted from initial) ± SEM (n=5). *$P < 0.05$ compared with mBSA/vehicle group.

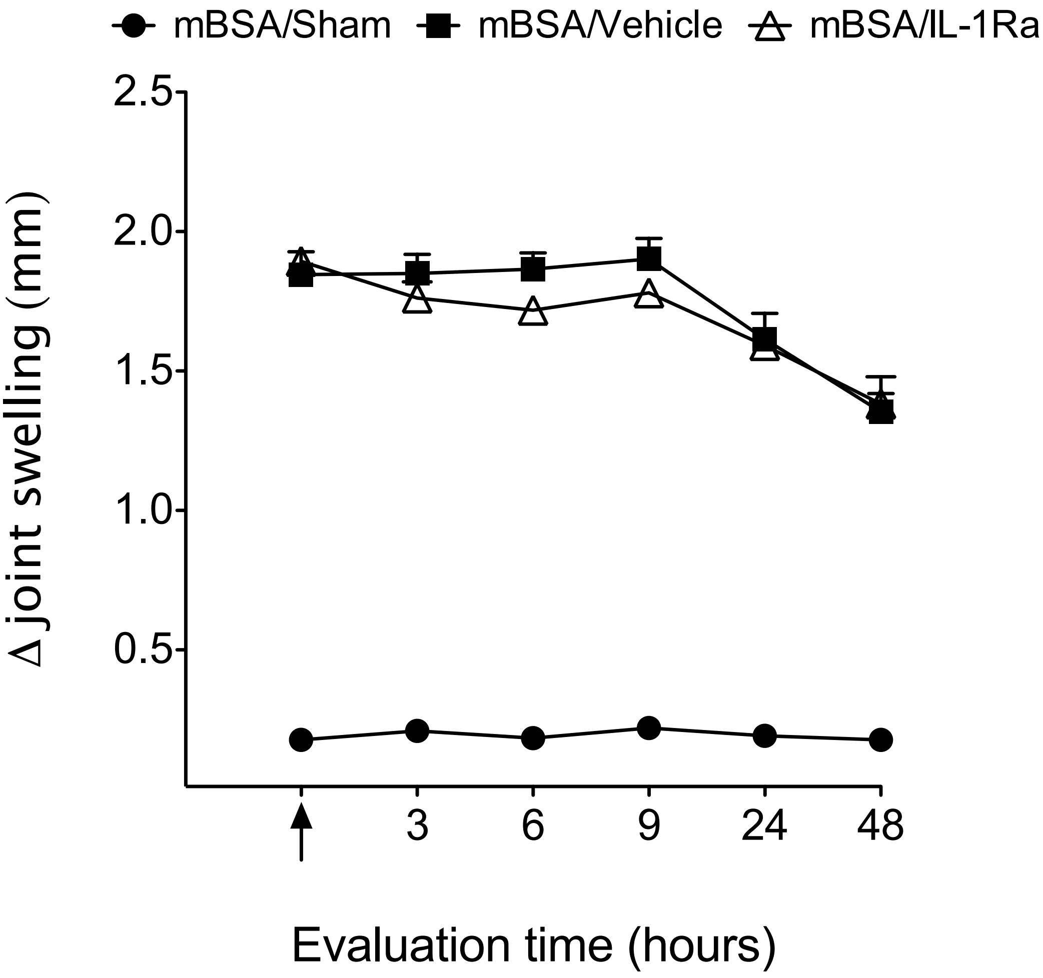

Figure S4- Effect of intrathecal injection of IL-1ra on joint swelling (oedema) in Balb/C mice during AIA model l. Immunized mice were challenged i.a. with 100 µg of mBSA or vehicle (veh - sterile saline). Animals were treated with IL-1Ra (300 ng/i.t.) 24 hours after mBSA challenge (indicate by arrow). Joint swelling was measure with a digital caliper in animals under anesthesia (isoflurane 2%) 2, 6 and 24 hours after drugs administration. Data are the means of delta (final measure subtracted from initial) ± S.E.M (n=5). *$P < 0.05$ compared with mBSA/vehicle group.
